# Glucose Metabolism Quantified by SUVmax on Baseline FDG-PET/CT Predicts Survival in Newly Diagnosed Multiple Myeloma Patients: Combined Harmonized Analysis of Two Prospective Phase III Trials

**DOI:** 10.3390/cancers12092532

**Published:** 2020-09-06

**Authors:** Anne-Victoire Michaud-Robert, Elena Zamagni, Thomas Carlier, Clément Bailly, Bastien Jamet, Cyrille Touzeau, Philippe Moreau, Françoise Kraeber-Bodere, Cristina Nanni, Caroline Bodet-Milin

**Affiliations:** 1 Nuclear Medicine Department, Nantes University Hospital, CRCINA INSERM, CNRS, Angers University, Nantes University, 44093 Nantes, France; annevictoire.michaud@chu-nantes.fr (A.-V.M.-R.); thomas.carlier@chu-nantes.fr (T.C.); clement.bailly@chu-nantes.fr (C.B.); bastien.jamet@chu-nantes.fr (B.J.) francoise.bodere@chu-nantes.fr (F.K.-B.); 2 Seragnoli Institute of Hematology, Bologna University School of Medicine, 40138 Bologna, Italy; e.zamagni@unibo.it; 3 Department of Hematology, Nantes University Hospital, CRCINA INSERM, CNRS, Angers University, Nantes University, 44093 Nantes, France; cyrille.touzeau@chu-nantes.fr (C.T.); philippe.moreau@chu-nantes.fr (P.M.); 4 Nuclear Medicine Department, ICO René Gauducheau, 44805 Saint-Herblain, France; 5 Nuclear Medicine, AOU Policlinico S.Orsola-Malpighi, 40138 Bologna, Italy; cristina.nanni@aosp.bo.it

**Keywords:** multiple myeloma, FDG-PET/CT, prognostic value, SUVmax

## Abstract

**Simple Summary:**

Multiple myeloma (MM) is associated with high morbidity and mortality and variable survival that requires early identification of high-risk patients in order to quickly adapt treatment. FDG-PET/CT is a promising technique for initial staging of symptomatic MM. The aim of our retrospective study was to asses the prognostic value of this technique at baseline in symptomatic MM patients included in two large European prospective studies. After harmonization of data by and ad-hoc approach called M-Combat, we confirmed the prognostic value of FDG-PET/CT in a population of 227 MM patients, by integrating a new prognostic biomarker named “bone SUVmax” (including the maximum intensity of fixation of focal lesions and bone marrow) which is strongly correlated with a poorer prognosis of MM patients. Prognostic patient stratification is currently based on laboratory tests and genomic abnormalities, but FDG-PET/CT is likely to be an important method of defining high-risk patients, and thus, to potentially better adapt future therapeutic management.

**Abstract:**

*Background:* Multiple myeloma is a hematological neoplasm characterized by a clonal proliferation of malignant plasma cells in the bone marrow, and is associated with high morbidity and mortality and variable survival. Positron emission tomography combined with computed tomography using 18F-deoxyfluoroglucose (FDG-PET/CT) is a promising technique for initial staging of symptomatic multiple myeloma patients. The objective of this study was to assess the prognostic value of this technique at baseline in symptomatic multiple myeloma patients included in two large European prospective studies (French and Italian). *Methods:* We retrospectively performed a combined harmonized analysis of 227 newly diagnosed transplant eligible multiple myeloma patients from two separate phase III trials. All images were centrally reviewed and analyzed using visual criteria and maximal standardized uptake value. An ad-hoc approach (called modified Combat) was applied to harmonize the data and then remove the “country effect” in order to strengthen the reliability of the final conclusions. *Results:* Using a multivariate analysis including treatment arm, R-ISS score, presence of extra-medullary disease and bone SUVmax, only bone SUVmax (*p* = 0.016) was an independent prognosis factor with an OS threshold of 7.1. For PFS, treatment arm and presence of extra-medullary disease were both independent prognosis biomarkers (*p* = 0.022 and 0.006 respectively). *Conclusions*: Our results show that bone SUVmax is a simple and reliable biomarker to analyze FDG-PET/CT at baseline that strongly correlates with a poorer prognosis for MM patients.

## 1. Introduction

Positron emission tomography using 18F-deoxyfluoroglucose (FDG-PET/CT) has been included in the 2019 International Myeloma Working Group (IMWG) recommendations as a feasible imaging strategy for the initial workup of newly diagnosed multiple myeloma (MM) patients [1]. FDG-PET/CT detects myeloma related lesions with excellent sensitivity and specificity [2] with the advantage of carrying out both bone and extra-bone exploration in a single examination. MM is associated with variable survival [3], and is due to intra- and inter-tumour heterogeneity. This demonstrates the potential benefits of identifying high-risk patients with poorer prognosis in order to adapt the treatment. Recognition of these high-risk patients is based on the identification of prognostic biomarkers including clinical variables, genomics and imaging results. Currently, four large prospective studies have shown prognostic value for FDG-PET/CT at baseline [2,4,5,6], but the results of these trials, although consistent, did not report the exact same prognostic biomarkers. Therefore, we sought to identify FDG-PET/CT prognostic biomarkers at diagnosis for a combined harmonized analysis of newly diagnosed transplant eligible (NTDE) MM patients enrolled in imaging sub-studies [2,5] of 2 independent European randomized phase III trials (IFM/DFCI2009 and EMN02/HO95) [7,8], in order to identify high risk patients.

## 2. Results

All 227 patients enrolled in these two studies were considered for this analysis. Fifty-four percent of patients were included in the autograft arm and 41% of patients in the medical treatment arm. Median follow-up was four years [range = 2 months to 6 years] with 121 events in progression free survival (PFS) and 48 in overall survival (OS). At diagnosis, 165 patients (73%) presented focal lesions (FLs) on FDG-PET/CT, and extra-medullary disease (EMD) was observed in 17 patients (7.5%), with no significant difference between the two studies. 

### 2.1. Before Harmonization

Before harmonization by M-Combat, bone marrow SUVmax (BM SUVmax) and bone SUVmax were significantly different between the two studies (*p* < 0.001 and *p* = 0.01 respectively). The patient characteristics are summarized in Table 1 and the characteristics of FDG-PET/CT parameters at baseline are summarized in Table 2.

### 2.2. After M-Combat Harmonization

**Figure 1:**
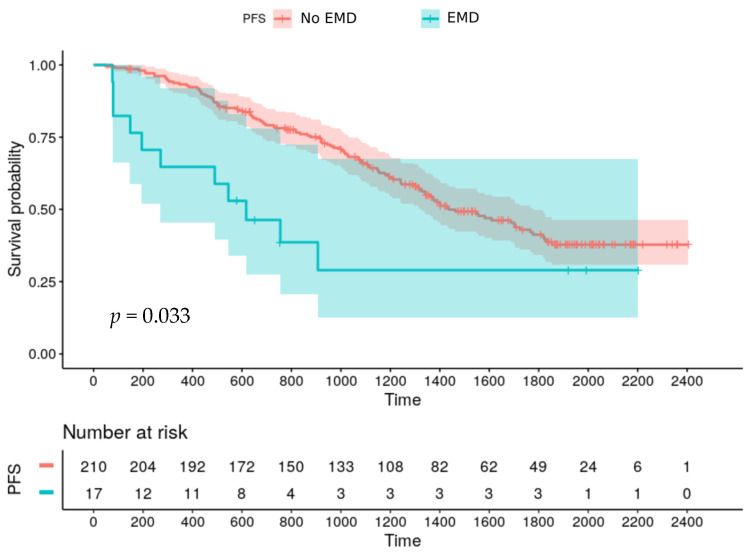
Progression free survival according to EMD at baseline.

After harmonization by M-Combat, univariate analysis revealed age and male gender negatively affect PFS (*p* = 0.035 and *p* = 0.40 respectively). Patients without EMD had a longer PFS compared to patients with EMD (median PFS: 48 months vs 20 months; *p* = 0.033) (Figure 1) but no significant difference was observed on OS.

**Figure 2:**
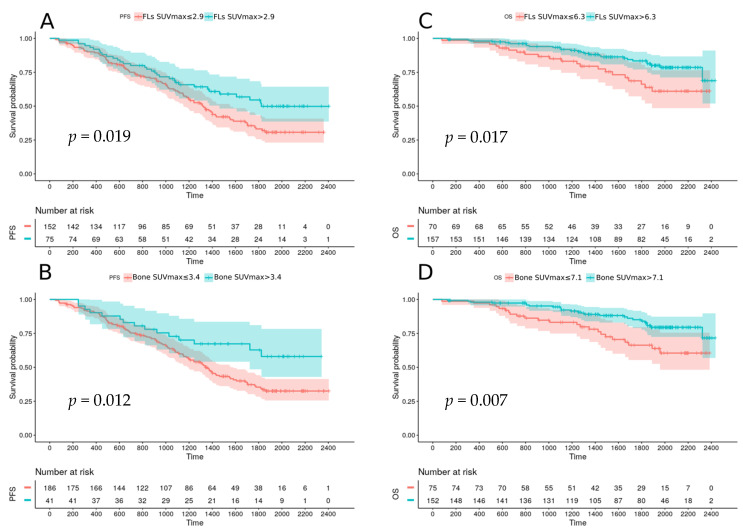
Progression free survival according to FL SUVmax (**A**) and Bone SUVmax (**B**) at baseline; Overall survival according to FL SUVmax (**C**) and bone SUVmax (**D**) at baseline.

The autograft treatment arm also had improved PFS compared to the medical arm (median PFS: 57 months vs 43 months; *p* = 0.038). Baseline FLs SUVmax and bone SUVmax significantly affected PFS and OS: PFS was shorter for patients with baseline FLs SUVmax higher than 2.9 and bone SUVmax higher than 3.4 (respectively, median PFS: 44 months vs 61 months, *p* = 0.019; 45 months vs not reached, *p* = 0.012) (Figure 2A,B). For OS, FLs SUVmax and bone SUVmax higher than 6.3 and 7.1 respectively were pejorative (*p* = 0.017 and *p* = 0.007 respectively) (Figure 2C,D).

**Figure 3:**
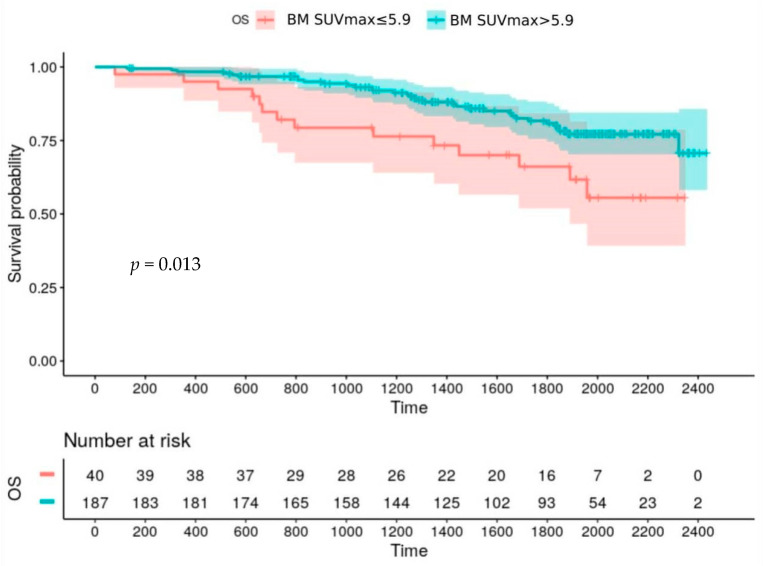
Overall survival according to BM SUVmax at baseline.

A baseline BM SUVmax higher than 5.9 also negatively affects OS (*p* = 0.013) (Figure 3).

Number of FLs and Deauville Score (DS) did not significantly affect survival. In a multivariate analysis including the treatment arm, R-ISS score, presence of EMD and bone SUVmax, absence of EMD and the autograft treatment arm were significantly and independently associated with a longer PFS, whereas bone SUVmax higher than 7.1 was the only prognostic parameter negatively affecting OS (Table 3).

## 3. Discussion

The present combined analysis demonstrated that reliable prognostic biomarkers can be extracted from baseline FDG-PET/CT data from a cohort of two populations after harmonization. To our knowledge this is the first time this has been performed in the context of multiple myeloma. The presence of EMD and intensity of tumour metabolism represented by bone SUVmax were strong predictors of unfavourable clinical outcomes. Both these factors, along with the treatment arm retained independent prognostic relevance in a multivariate analysis, on PFS for EMD and treatment arm and on OS for bone SUVmax. 

While the prognostic impact of EMD has already been demonstrated in large prospective studies [2,5,6] reflecting the dedifferentiation of the disease, FDG absorption had only been demonstrated in 2011 by the Italian group with a FL SUVmax FDG threshold of 4.2 [5]. Our study confirms the prognostic potential of SUVmax by integrating the concept of bone SUVmax with a threshold of 7.1. This threshold value is higher than that presented by Zamagni et al., and is likely due to more modern and effective treatments. This biomarker is an easily measured parameter in standard clinical practice. Its value includes FLs SUVmax, reflecting tumor metabolism and possibly interactions between plasma cells and environmental bone cells, and BM SUVmax also reflecting tumor activity as well as contributions from a reactive environment (anaemia, inflammation). It is thus the combined analysis of all of these biological parameters, which through bone SUVmax seems to have the most robust prognostic value. We did not observe any impact of the DS on patient survival at baseline, contrary to therapy assessment as demonstrated by another combined study [9]. 

Our study completes and proves once again the essential role of this examination in the diagnosis of MM [10] which is also recommended for therapeutic evaluation [1]. In addition, MM is a pathology with very varied risk profiles and very expensive therapeutic approaches. It will be important in the near future to rapidly define the patient’s risk group in order to better orient the therapeutic strategy in this context, PET imaging, unlike biological and genomic tests, allows a sensitive analysis of the disease at the whole body level, with results that can be obtained quickly and at a limited cost. Moreover, FDG-PET/CT is the only modality that can provide a whole body representation of the heterogeneity of the disease [11]. Imaging data from glucose metabolism analyses are probably correlated with certain biological data (e.g., hexokinase and FDG-PET/CT negativity [12]) but it is also thought that they may be complementary, as FDG uptake may reflect tumor activity but also the interactions of the tumor and its environment. We believe that in view of the prognostic impact of the biomarkers identified in our study, which are consistent with the prospective multicenter studies already published, we could consider integrating the PET-FDG biomarkers (bone SUVmax and EMD) into a prognostic score such as the R-ISS score integrating biological, and high-risk cytogenetic abnormalities recognized as negatively affecting patient prognosis [13]. Radiomics data may also, in the coming months, increase this prognostic score [14] and correlation between FDG-PET/CT and genomics is ongoing in the Cassiopet study [6]. 

The major finding of our study is to participate in the harmonization of the interpretation of baseline FDG-PET/CT data by confirming the values of the EDM lesions and also the LF and BM SUV to determine the SUVmax bone. Nevertheless, this is a retrospective analysis whose results, particularly the definition and threshold of the bone SUVmax, needs to be studied and validated by prospective studies. 

## 4. Materials and Methods

### 4.1. Patients

In the present analysis, we included 227 NDTE MM patients enrolled in the imaging sub-studies of trials IFM/DFCI2009 (134 patients) and EMN02/HO95 (93 patients). The French multicentric prospective study IFM/DFCI2009 evaluated the combination of 8 cycles of Lenalidomide, Bortezomib and Dexamethasone (RVD) versus RVD plus autologous stem cell transplantation (ASCT) followed for all patients by Lenalidomide maintenance [8,15]. The imaging sub-study called IMAJEM (IMAgerie JEune Myélome) compared axial MRI and whole body FDG-PET/CT at diagnosis, after 3 cycles of induction therapy and before maintenance. The primary endpoint was the comparison of bone lesion detection rate at diagnosis by MRI and FDG-PET/CT, and the secondary endpoint was the prognostic impact of both imaging at diagnosis, after 3 cycles of induction therapy and before maintenance therapy [2] (for further details, please refer to ClinicalTrials.gov, NCT01309334).

The Italian multicentric prospective study EMN02/HO95 compared ASCT versus proteasome inhibitor based intensification, and consolidation therapy versus no consolidation, followed for all patients by Lenalidomide maintenance [8]. The imaging sub-study evaluated whole-body FDG-PET/CT at diagnosis, after induction therapy and before maintenance, with the aim of determining the prognostic significance of FDG-PET/CT at diagnosis after therapy, and secondly to standardize FDG-PET/CT evaluation and to then define interpretation criteria [5] (for further details, please refer to ClinicalTrials.gov, NCT01134484).

All patients gave informed consent. Both studies were approved by local ethics committees (CPP Ouest II-Angers and CPP reference 2010-32, Eudra CT NCT01309334, n°IDRCB: 2010-A01382-37, ANSM autorisation: 18 January 2011) and registered at ClinicalTrials.gov (NCT01309334 and NCT01134484, respectively).

### 4.2. FDG-PET/CT Evaluation

FDG-PET/CT images were acquired in each center according to the local protocol. All centers involved in the trials used the same generation scanners and followed the rules of good practice defined for PET imaging in oncology [16].

More specifically, all French-center patients fasted for at least 4 hours before FDG injection. Blood glucose levels measured before administering FDG preferably had to be < or =150 mg/dL, but < or =200 mg/dL was allowed. No insulin was administered before the FDG injection. Water was given orally during the FDG uptake phase. Whole-body imaging (top of the head to the feet, arms alongside the body) was performed 60 to 80 minutes after the intravenous injection of 3 to 7 MBq/kg FDG. For Italian-center patients, all patients fasted for at least 6 hours before FDG injection. Blood glucose levels measured before administering FDG preferably had to be < or =200 mg/dL. No insulin was administered before the FDG injection. Extended Total-body imaging (top of the head to femurs, arms alongside the body) was performed 60 ± 10 minutes after the intravenous injection of 3 to 5 MBq/kg FDG. For each French or Italian examination, the low-dose CT was immediately followed by PET acquisition.

The French and Italian FDG-PET/CT baseline images were reread on a dedicated workstation (Imagys, Keosys, France) by nuclear physicians of the Nantes University Hospital (AVMR, CBM, FKB). The following interpretation criteria were reported:

- Number, location and SUVmax of FLs, defined as the presence of areas of focally increased tracer uptake on bone, with or without any underlying lytic lesion on CT, and present on at least two consecutive slices (excluding uptake in relation to osteoarticular benign pathologies.
- Number, location and SUVmax of EMD defined as tracer uptake on tissue not contiguous to bone.
- SUVmax of BM, measured at the lumbar vertebrae (L3-L5) excluding focal lesions with a 3D rectangular ROI

FDG uptake degree of the hottest FL (or BM in cases without FL) was visually quantified with the 5-point DS [17]. Semi-quantitative measures were obtained in physiological areas corresponding to reference organs, liver (SUVmax) and mediastinal blood pool (SUVmax). Furthermore, we reported the highest SUVmax on bone analysis, including FLs and BM uptake, called bone SUVmax.

### 4.3. Laboratory Investigations

Blood cell count, serum levels of lactate dehydrogenase (LDH), albumin and β2microglobulin were evaluated before treatment. BM aspirate was evaluated at baseline and FISH analysis of del(17p), t(4;14), t(14;16) was performed.

### 4.4. Statistical Analysis

All clinico-biological characteristics and FDG-PET/CT parameters of the two populations were merged and compared. Next, harmonization of FDG-PET/CT semi-quantitative parameters based on SUV was performed using the modified Combat method (M-Combat) with the aim of removing the “country effect” [18]. Briefly, the M-Combat approach aims to address the problem of feature variability due to inherent heterogeneity of acquisition set-up directly related to the final value of the feature considered. This problem could be solved within an empirical Bayes framework by finding a transformation to express all features in a common space so as to avoid the “country effect”. The M-Combat method shifts the values to a centered reference rather than an arbitrary space typically related to the mean and variance of the features. This approach recently proved its usefulness in the context of locally advanced cervical cancer and locally advanced laryngeal cancer using biomarkers extracted from FDG-PET [19]. A maximally selected Rank Statistics method was adopted to find the prognostic threshold of FDG-PET/CT characteristics. Progression-free survival (PFS) was defined as the time from randomization to the first documentation of progressive disease or to death from any cause. Survival rates for the groups of patients were calculated using the Kaplan-Meier method and curves were compared using the exact log-rank test. Univariate prognostic analyses for PFS and OS were compared with the Cox proportional-hazards model including the following variables: age, sex, revised International Staging System (R-ISS), treatment arm, presence of EMD, number of FLs, FLs SUVmax, BM SUVmax, FLs DS, BM DS and bone SUVmax, and then, a multivariate analysis was performed. *p* values < 0.05 were considered significant.

## 5. Conclusions

In conclusion, our study showed that bone SUVmax is a simple and reliable way of interpreting baseline FDG-PET/CT and strongly correlated with a poorer prognosis of MM patients. Whilst prognostic patient stratification is currently based on laboratory tests and genomic abnormalities, FDG-PET/CT functional imaging is likely to be an important method of defining high-risk patients, and thus, to potentially better adapt future therapeutic management. 

## Figures and Tables

**Table 1:** Patient characteristics at baseline.

Characteristic (*n* Patients)		Overall (227)	France (134)	Italy (93)	*p*-Value
**Median Age (IQR)**		59 (53, 62)	59 (53, 62)	58 (52, 62)	0.573
**Random Assignment (%)**	- **Bortezomib Intensification** - **Autograft** - **Missing Data**	94 (41.4) 124 (54.6) 10 (4.0)	72 (53.7) 61 (45.5) 1 (0.8)	22 (23.7) 63 (67.7) 8 (8.6)	< 0.001
**ISS *n* (%)**	- **Stage I** - **Stage II** - **Stage III**	102 (45.2) 89 (39.0) 36 (15.8)	57 (42.5) 57 (42.5) 20 (14.9)	45 (48.9) 32 (34.0) 16 (17.0)	0.424
**R-ISS *n* (%)**	- **Stage I** - **Stage II** - **Stage III** - **Missing Data**	54 (23.7) 124 (54.7) 24 (10.6) 25 (11.0)	27 (20.2) 74 (55.2) 14 (10.4) 19 (14.2)	27 (29.1) 50 (53.7) 10 (10.7) 6 (6.5)	0.135
**LDH (U/L) (median (IQR))**		31.00 (166, 337)	211.80 (159, 327)	263.50 (179, 365)	0.093
**High Risk Cytogenetics FISH** **N (%) (del(17p), t(4;14), t(14;16))**		6 (14.0)	11 (10.7)	15 (18.1)	0.202
**β_2_m mg/L (median (IQR))**		3.20 (2.40, 4.45)	3.25 (2.61, 4.48)	3.10 (2.21, 4.38)	0.405
**Albumin g/dL (median (IQR))**		3.86 (3.45, 4.26)	3.77 (3.39, 4.23)	3.95 (3.50, 4.40)	0.034
**Platelets (median (IQR))**		232.00 (189.5, 282.5)	233.00 (194.0, 279.0)	225.50 (176.3, 282.8)	0.400

ISS: International Staging System; R-ISS: revised International Staging System, LDH: Lactate dehydrogenase; β2m: β2microglobulin.

**Table 2:** Characteristics of FDG-PET/CT parameters at baseline (before harmonization).

Baseline (*n* Patients)	Overall (227)	France (134)	Italy (93)	*p*-Value
**Presence of FLs *n* (%)**	165 (72.7)	99 (73.9)	66 (70.9)	0.652
**Presence of EMD *n* (%)**	17 (7.5)	13 (9.7)	4 (4.3)	0.199
**BM SUVmax (Median [IQR])**	3.40 [2.63, 4.50]	3.70 [2.90, 4.97]	2.82 [2.29, 3.82]	< 0.001
**FL SUVmax (Median [IQR])**	5.60 [4.0, 8.5]	5.70 [4.45, 8.45]	5.34 [3.59, 8.56]	0.306
**Liver SUVmax (Median [IQR])**	3.27 [2.79, 3.87]	3.28 [2.80, 3.90]	3.26 [2.52, 3.86]	0.248
**Mediastinal SUVmean (Median [IQR])**	1.56 [1.33, 1.80]	1.54 [1.34, 1.72]	1.58 [1.27, 1.86]	0.768
**Bone SUVmax (Median [IQR])**	5.00 [3.45, 7.89]	5.20 [3.8, 8.00]	4.30 [1.06, 7.44]	0.01

FLs: focal lesions; BM: bone marrow; EMD: extra-medullary disease; SUV: standard uptake value.

**Table 3:** Univariate and multivariate analysis of baseline variables on PFS and OS.

Variable	HR	95% CI		*p*
**PFS**				
**Univariate Analysis**				
Age	1.562	1.031	2.365	**0.035**
Sex	1.478	1.017	2.148	**0.040**
R-ISS 1 vs 2-3	1.217	0.813	1.847	0.354
R-ISS 1-2 vs 3	1.504	0.873	2.589	0.141
Autograft Arm	0.683	0.475	0.982	**0.039**
FL DS 1-2-3 vs 4-5	1.098	0.756	1.594	0.622
BM DS 1-2-3 vs 4-5	1.180	0.816	1.706	0.380
FLs Number < 1*	0.868	0.503	1.172	0.221
Presence of EMD	2.324	1.246	4.335	0.008
FLs SUVmax ≤ 2.9*	0.634	0.424	0.946	0.026
Bone SUVmax ≤ 3.4*	0.528	0.307	0.907	0.021
BM SUVmax ≤ 3.3*	0.720	0.499	1.041	0.081
**Multivariate Analysis**				
Presence of EMD	2.510	1.297	4.869	**0.006**
Autograft Arm	0.640	0.442	0.938	**0.022**
**OS**				
**Univariate Analysis**				
Age	0.591	0.322	1.089	0.091
Sex	1.108	0.621	1.978	0.728
R-ISS 1 vs 2-3	1.313	0.669	2.575	0.428
R-ISS 1-2 vs 3	1.836	0.857	3.930	0.118
Autograft Arm	1.051	0.727	1.518	0.792
FL DS 1-2-3 vs 4-5	1.139	0.630	2.0.58	0.666
BM DS 1-2-3 vs 4-5	1.457	0.823	2.577	0.196
FLs Number < 1*	0.521	0.244	1.115	0.093
Presence of EMD	2.107	0.894	4.965	0.088
FLs SUVmax ≤ 6.3*	0.501	0.283	0.887	0.018
Bone SUVmax ≤ 7.1*	0.462	0.262	0.814	0.007
BM SUVmax ≤ 5.9*	0.450	0.241	0.840	0.012
**Multivariate Analysis**				
Bone SUVmax > 7.1*	2.020	1.140	3.592	**0.016**

R-ISS: revised International Staging System; EMD: extra-medullary disease; FLs: focal lesions; BM: bone marrow; SUV: standard uptake value; DS: Deauville score; *optimum threshold defined by Maximally selected Rank Statistics method.
